# Tumor necrosis is an important hallmark of aggressive endometrial cancer and associates with hypoxia, angiogenesis and inflammation responses

**DOI:** 10.18632/oncotarget.5344

**Published:** 2015-10-14

**Authors:** Geir Bredholt, Monica Mannelqvist, Ingunn M. Stefansson, Even Birkeland, Trond Hellem Bø, Anne M. Øyan, Jone Trovik, Karl-Henning Kalland, Inge Jonassen, Helga B. Salvesen, Elisabeth Wik, Lars A. Akslen

**Affiliations:** ^1^ Centre for Cancer Biomarkers CCBIO, Department of Clinical Medicine, Section for Pathology, University of Bergen, Bergen, Norway; ^2^ Department of Pathology, Haukeland University Hospital, Bergen, Norway; ^3^ CCBIO, Department of Informatics, University of Bergen, Bergen, Norway; ^4^ Department of Microbiology, Haukeland University Hospital, Bergen, Norway; ^5^ Center for Cancer Biomarkers CCBIO, Department of Clinical Science, University of Bergen, Norway; ^6^ Department of Gynecology and Obstetrics, Haukeland University Hospital, Bergen, Norway

**Keywords:** necrosis, hypoxia, angiogenesis, inflammation, gene signatures

## Abstract

**Aims:**

Tumor necrosis is associated with aggressive features of endometrial cancer and poor prognosis. Here, we investigated gene expression patterns and potential treatment targets related to presence of tumor necrosis in primary endometrial cancer lesions.

**Methods and Results:**

By DNA microarray analysis, expression of genes related to tumor necrosis reflected multiple tumor-microenvironment interactions like tissue hypoxia, angiogenesis and inflammation pathways. A tumor necrosis signature of 38 genes and a related patient cluster (Cluster I, 67% of the cases) were associated with features of aggressive tumors such as type II cancers, estrogen receptor negative tumors and vascular invasion. Further, the tumor necrosis signature was increased in tumor cells grown in hypoxic conditions *in vitro*. Multiple genes with increased expression are known to be activated by HIF1A and NF-kB.

**Conclusions:**

Our findings indicate that the presence of tumor necrosis within primary tumors is associated with hypoxia, angiogenesis and inflammation responses. HIF1A, NF-kB and PI3K/mTOR might be potential treatment targets in aggressive endometrial cancers with presence of tumor necrosis.

## INTRODUCTION

Tumor hypoxia is an important feature of aggressive cancers [1], and as a morphologic marker, necrosis is known to be associated with poor prognosis in a variety of tumors [2–5]. In endometrial cancer, necrosis is related to increased tumor cell proliferation, high FIGO stage, and reduced disease-specific survival [6–8]. We previously found that necrosis was associated with activated angiogenesis, reduced vascular maturation and presence of vascular invasion, suggesting a link between necrosis, tumor-vascular interactions and metastatic spread [9]. In breast tumors, necrosis has been related to high-grade disease, increased tumor size, estrogen receptor negative status, high microvessel density, and macrophage infiltration [3, 10, 11].

Hypoxic tumor cells surrounding necrotic tissue may represent areas of dedifferentiation and development or selection of increasingly malignant cells [12]. In tumors, inflammatory cells tend to accumulate within necrotic foci [13], and degraded tumor cells are known to release pro-inflammatory cytokines that may stimulate angiogenesis and cancer progression [14]. Further, angiogenic factors are secreted by tumor associated macrophages in such areas [15]. It appears that hypoxia promote high-grade tumor features [16], and necrosis has been associated with tumor progression and increased resistance to radiation and chemotherapy [17, 18].

Here, we explored differential gene expression patterns and potential treatment targets associated with tumor necrosis in endometrial cancers. Our findings indicate that necrosis and tumor gene expression patterns are associated with hypoxia, inflammation and angiogenesis networks.

## RESULTS

### Tumor necrosis is associated with aggressive clinicopathologic features and reduced patient survival

**Figure 1:**
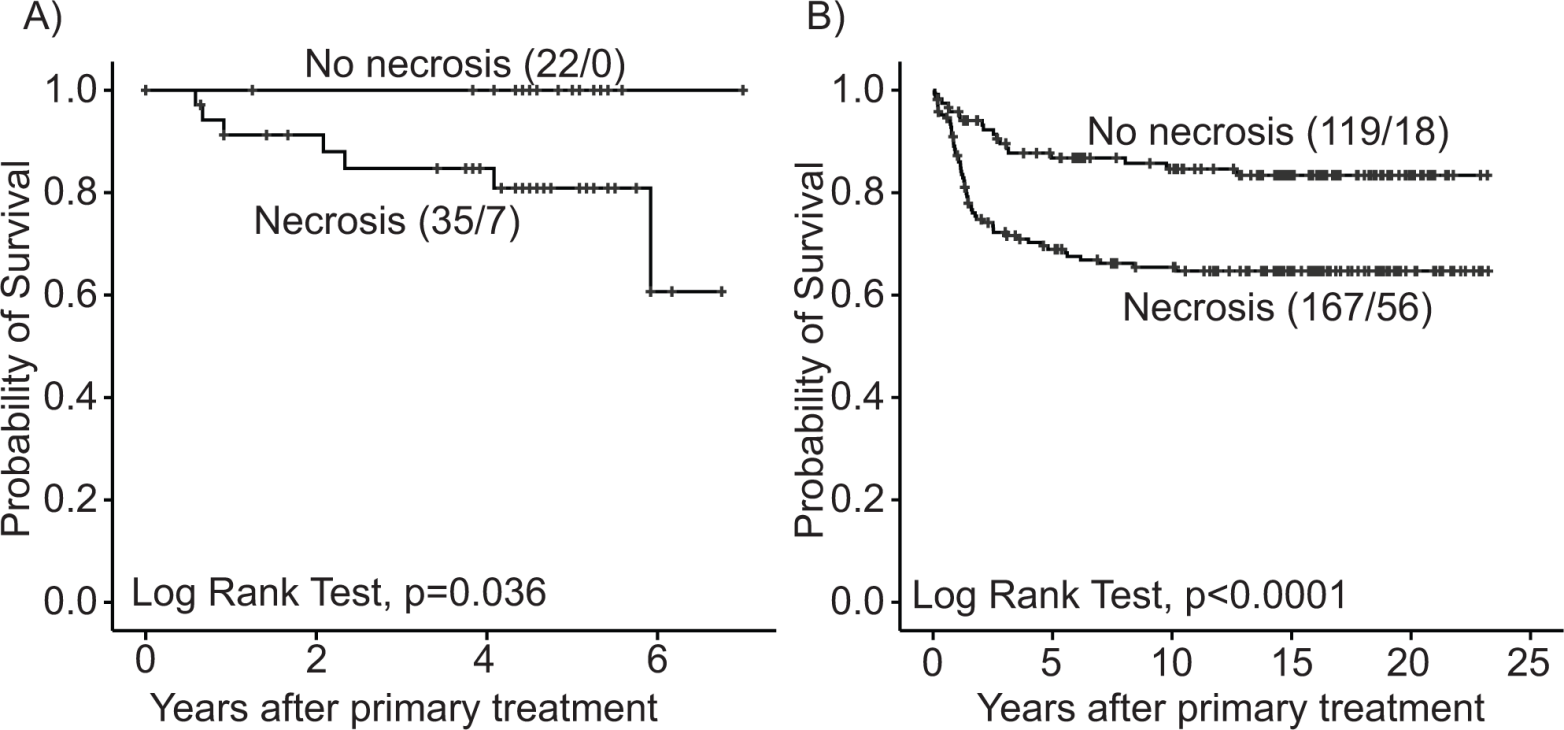
Estimated survival among endometrial carcinoma patients according to tumor necrosis **A.** Series I (*N* = 57) and **B.** Series II (*N* = 286), for each category, total number of cases/events is given.

In Series I (*n* = 57), tumor necrosis (present in 61% of the tumors) was associated with aggressive features such as non-endometrioid subtype, histologic grade 3 and FIGO stage III/IV (Table 1). Presence of necrosis was correlated to vascular invasion and to the tumor subgroup classified as aggressive by a gene expression signature published by Salvesen et al. [19] In Series II (*n* = 286), tumor necrosis (present in 58%) was associated with similar aggressive features and advanced stage (Table 1), and necrosis was associated with decreased patient survival in both series (Figure 1). No significant differences were found between Series I and II with respect to distribution of basic clinicopathologic factors (data not shown).

**Table 1:** Presence or absence of necrosis in Series I (*N* = 57) and II (*N* = 286) and associations with clinico-pathologic features

		Series I			Serie II		
		Necrosis − *n* (%)	Necrosis + *n* (%)	*p*-value*	Necrosis − *n* (%)	Necrosis + *n* (%)	*p*-value*
Histologic type	Endometrioid	22 (43)	29 (57)	0.04	114 (44)	143 (56)	0.005
	Non-endometrioid	0 (0)	6 (100)		5 (17)	24 (83)	
Histologic grade	Grade 1 and 2	21 (48)	23 (52)	0.009	98 (55)	79(45)	< 0.0001
	Grade 3	1 (8)	12 (92)		21 (19)	88 (81)	
Type II cancer^¶^ [77]	No	21 (52)	19 (48)	0.001	-	-	-
	Yes	1 (6)	16 (94)		-	-	
Estrogen receptor^†^	Positive	21 (50)	21 (50)	0.003	65 (52)	59 (48)	0.0005
	Negative	1 (7)	14 (93)		44 (31)	99 (69)	
Progesterone receptor^†^	Positive	20 (44)	25 (56)	0.079	49 (53)	44 (47)	0.011
	Negative	2 (17)	10 (83)		62 (36)	108 (64)	
Mitoses^‡^	Low	19 (45)	23 (55)	NS	105 (49)	111 (51)	< 0.001
	High	3 (20)	12 (80)		14 (20)	56 (80)	
Vascular invasion	Absent	20 (57)	15 (43)	< 0.001	95 (52)	88 (48)	0.013
	Present	2 (9)	20 (91)		24 (23)	79 (77)	
Myometrial infiltration	< 50%	15 (50)	15 (50)	0.062	56 (48)	61 (52)	0.004
	≥ 50%	7 (29)	20 (71)		22 (26)	62 (74)	
FIGO stage	I/II	22 (46)	26 (54)	0.01	16 (29)	39 (71)	0.039
	III/IV	0 (0)	9 (100)		102 (44)	128 (56)	
Aggressive cluster [78]	No	16 (55)	13 (45)	0.009	-	-	-
	Yes	6 (21)	22 (79)		-	-	

* Chi-sq uare test, two-sided

† Cut-point median

‡ Cut-point upper quartile

¶ Type I cancers are highly and moderately differentiated tumors, have superficial invasion of the myometrium, have high sensitivity to progestogens and favorable prognosis. Type II cancers are poorly differentiated tumors, have deeper invasion of tumor into the myometrium, higher frequency of metastatic spread into the pelvic lymph nodes, decreased sensitivity to progestogens and less favorable prognosis

### Tumor necrosis and associated gene expression patterns

**Figure 2:**
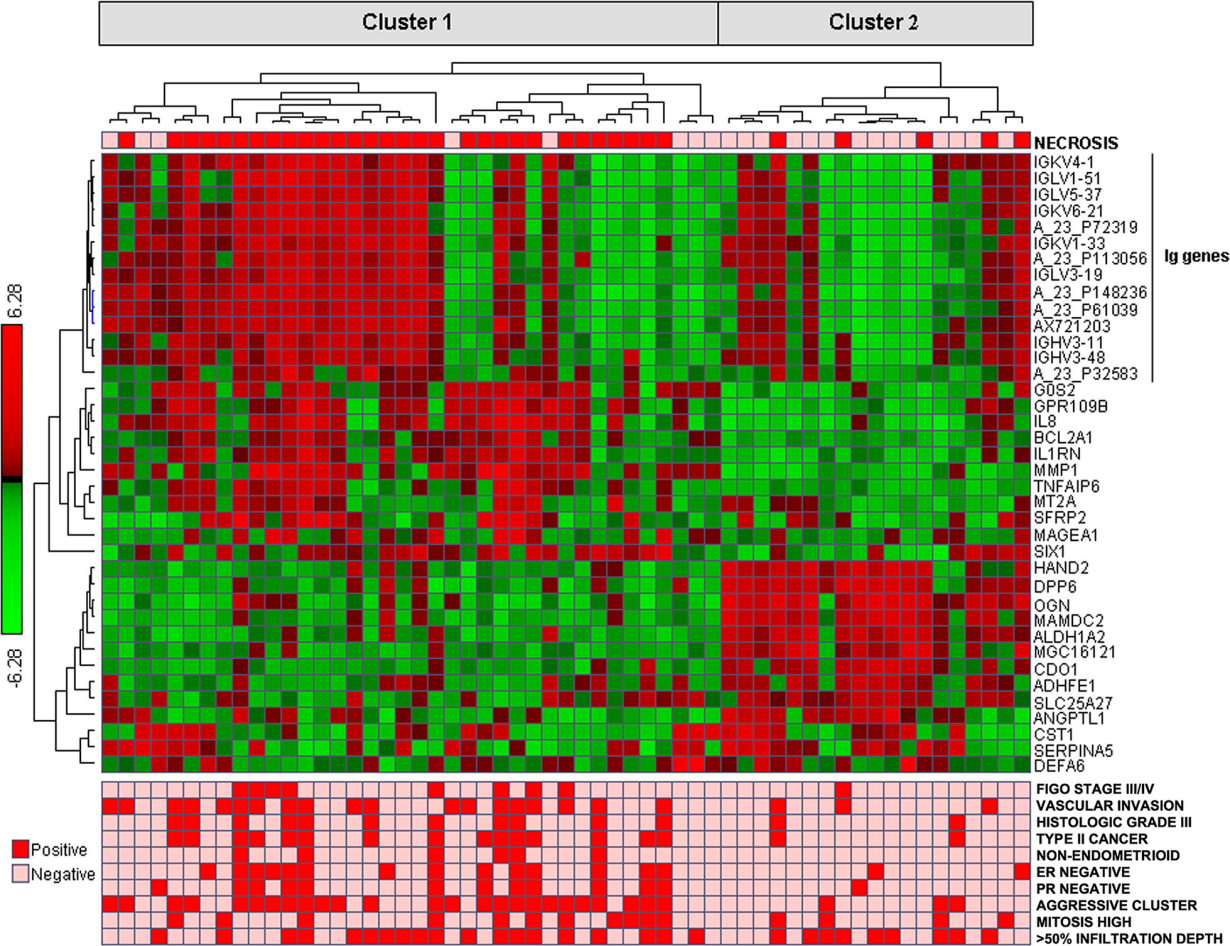
Cluster diagram of genes from SAM analysis; 38 genes differentially expressed between necrosis+ and necrosis-tumors In the lower part of the panel, related clinico-pathologic characteristics are shown

Analysis by SAM with respect to tumor necrosis indicated that 25 genes were significantly up-regulated and 13 genes down-regulated in cases with presence of necrosis (fold change ≥ 2; false discovery rate 0.06) (Table 2). 10 of these genes generated from microarray analysis were validated by qPCR. The two methods showed strongly and significantly correlated expression values (data not shown). From hierarchical clustering of these 38 genes (Pearson correlation as similarity measure, average linkage WPGMA), one of the two main clusters were significantly associated with tumor necrosis (*p* = 0.0001) as well as other aggressive clinico-pathological characteristics (Supplementary Table S1, Figure 2). High tumor necrosis signature (TNS) (by median value) showed a trend for reduced survival, *p* = 0.058 (Supplementary Figure S1).

**Table 2:** Genes differentially expressed in samples with tumor necrosis (*n* = 35) compared with non-necrotic tumors (*n* = 22) tissues

Gene symbol	Gene name	Fold Change
**Up-regulated genes**		
MMP1	Matrix metallopeptidase 1 (interstitial collagenase)	7.2
SFRP2	Secreted frizzled-related protein 2	5.6
IGLV1-51	Immunoglobulin lambda variable 1–51	4.1
CXCL8^P^	Chemokine (C-X-C motif) ligand 8	3.8
MAGEA1	Melanoma antigen family A1	3.3
IGKV4-1	Immunoglobulin kappa variable 4–1	3.0
TNFAIP6^P^	Tumor necrosis factor, alpha-induced protein 6	3.0
IGKV6-21	Immunoglobulin kappa variable 6–21	3.0
HCAR3^P^	Hydroxycarboxylic acid receptor 3	2.7
IL1RN	Interleukin 1 receptor antagonist	2.6
IGLV5-37	Immunoglobulin lambda variable 5–37	2.6
IGHA1	Immunoglobulin heavy constant alpha 1	2.6
A_23_P113056		2.5
MT2A^P^	Metallothionein 2A	2.5
A_23_P32583		2.5
BCL2A1^P^	BCL2-related protein A1	2.4
SIX1^P^	SIX homeobox 1	2.4
A_23_P84795		2.4
IGLV3-19	Immunoglobulin lambda variable 3–19	2.3
IGHA1	Immunoglobulin heavy constant alpha 1	2.3
IGKV1-33	Immunoglobulin kappa variable 1–33	2.3
A_23_P61039		2.3
G0S2	G0/G1switch 2	2.2
IGHV3-11	Immunoglobulin heavy variable 3–11	2.1
IGHV3-48^P^	Immunoglobulin heavy variable 3–48	2.1

**Down-regulated genes**		
DEFA6	defensin, alpha 6, Paneth cell-specific	− 9.2
CST1^P^	Cystatin SN	− 3.3
OGN^P^	Osteoglycin (osteoinductive factor, mimecan)	− 3.0
SERPINA5^P^	Serpin peptidase inhibitor, clade A (alpha-1 antiproteinase, antitrypsin), member 5	− 2.8
MAMDC2	MAM domain containing 2	− 2.7
ANGPTL1	Angiopoietin-like 1	− 2.6
ALDH1A2^P^	Aldehyde dehydrogenase 1 family, member A2	− 2.4
DPP6	Dipeptidyl-peptidase 6	− 2.3
MIR503HG	MIR503 host gene (non-protein coding)	− 2.2
HAND2	Heart and neural crest derivatives expressed 2	− 2.2
SLC25A27^P^	Solute carrier family 25, member 27	− 2.0
ADHFE1^P^	Alcohol dehydrogenase, iron containing, 1	− 2.0
CDO1	Cysteine dioxygenase, type I	− 2.0

*P* = predictor genes

Using the 38 SAM genes in a diagonal linear discriminant predictor, the correct classification rate by LOOCV was 75%. Using a condensed and fixed gene subset of 13 of the 38 genes as predictor (7 up-regulated and 6 down-regulated genes marked in Table 2) gave 87% correct prediction.

### Tumor necrosis is associated with markers of hypoxia

Gene set enrichment analysis (GSEA) indicates tumor necrosis to be associated with several data sets related to hypoxia, angiogenesis and inflammation (Table 3). Among genes differentially expressed between tumor samples with and without necrosis, a substantial number of hypoxia-related genes were found to be up-regulated, such as IL6, CXCL8 (IL8), SERPINE1 as well as genes involved in glycolysis (SLC2A3, LDHA) (Table 2 and Supplementary Table S2). Also, gene ontology (GO) analysis showed significant enrichment of the biological process *glycolysis* (*p* < 0.0001). Several hypoxia-induced genes are included in this GO category (data not shown).

**Table 3:** GSEA analysis showing gene sets related to the themes hypoxia, angiogenesis, inflammation, prognosis and ER status

Description of gene set	Gene set size	Genes enriched	FDR (%)
**Gene sets related to hypoxia**			
Combination of all hypoxia target gene sets in the GSEA MSigDB database*	152	67 (44%)	5.6
Genes upregulated in hypoxic macrophages [79]	30	13 (43%)	6.5
GO:0006096 and GO:0019642: Glycolysis (homo sapiens)^†^	36	17 (47%)	7.1
Genes upregulated in hypoxic epithelial cells [80]	51	23 (45%)	7.9
Gene expression signature of melanoma spheroids with a necrotic core compared to monolayer culture [81]	72	44 (61%)	8.1
Genes involved in glycolysis^¶^	29	17 (51%)	9.1
**Gene sets related to angiogenesis**			
Extracellular matrix molecules related to endothelial cell activation^‡^	33	12 (36%)	6.2
HUVEC stimulated with a combination of HGF and VEGF [82]	178	75 (42%)	7.8
Genes upregulated by EGFR1^§^	135	74 (55%)	9.5
Genes upregulated by TGF-β^§^	646	214 (33%)	17.0
**Gene sets related to stress and inflammatory processes**			
NF-κB genes and confirmed targets [83]	46	24 (52%)	5.8
Human genes with confirmed promoter binding sites for NF-κB^║^	77	39 (51%)	6.5
Genes upregulated by IL1^§^	132	59 (45%)	6.9
Genes upregulated by IL1B in human endometrial stromal cells [84]	17	14 (82%)	7.3
Genes upregulated by IL5^§^	108	43 (40%)	7.3
Confirmed NF-κB target genes^††^	185	83 (45%)	8.0
**Gene sets related to prognosis and estrogen receptor**			
Genes upregulated in breast cancers with poor prognosis [85]	129	64 (50%)	6.1
70 gene signature associated with poor prognosis in breast cancer [85]	45	23 (51%)	6.2
Genes upregulated in estrogen receptor negative breast cancers [85]	878	413 (47%)	11.4
Human estrogen responsive genes [86]	781	263 (34%)	16.8

Gene sets in the different groups are ranked according to the false discovery rate (FDR)

* http://www.broad.mit.edu/gsea/msigdb/

† http://amigo.geneontology.org/

¶ http://www.pantherdb.org/

‡ http://www.superarray.com

§ http://www.netpath.org

║ http://bioinfo.lifl.fr/NF-KB/

†† http://www.bu.edu/nf-kb/gene-resources/target-genes/

**Figure 3:**
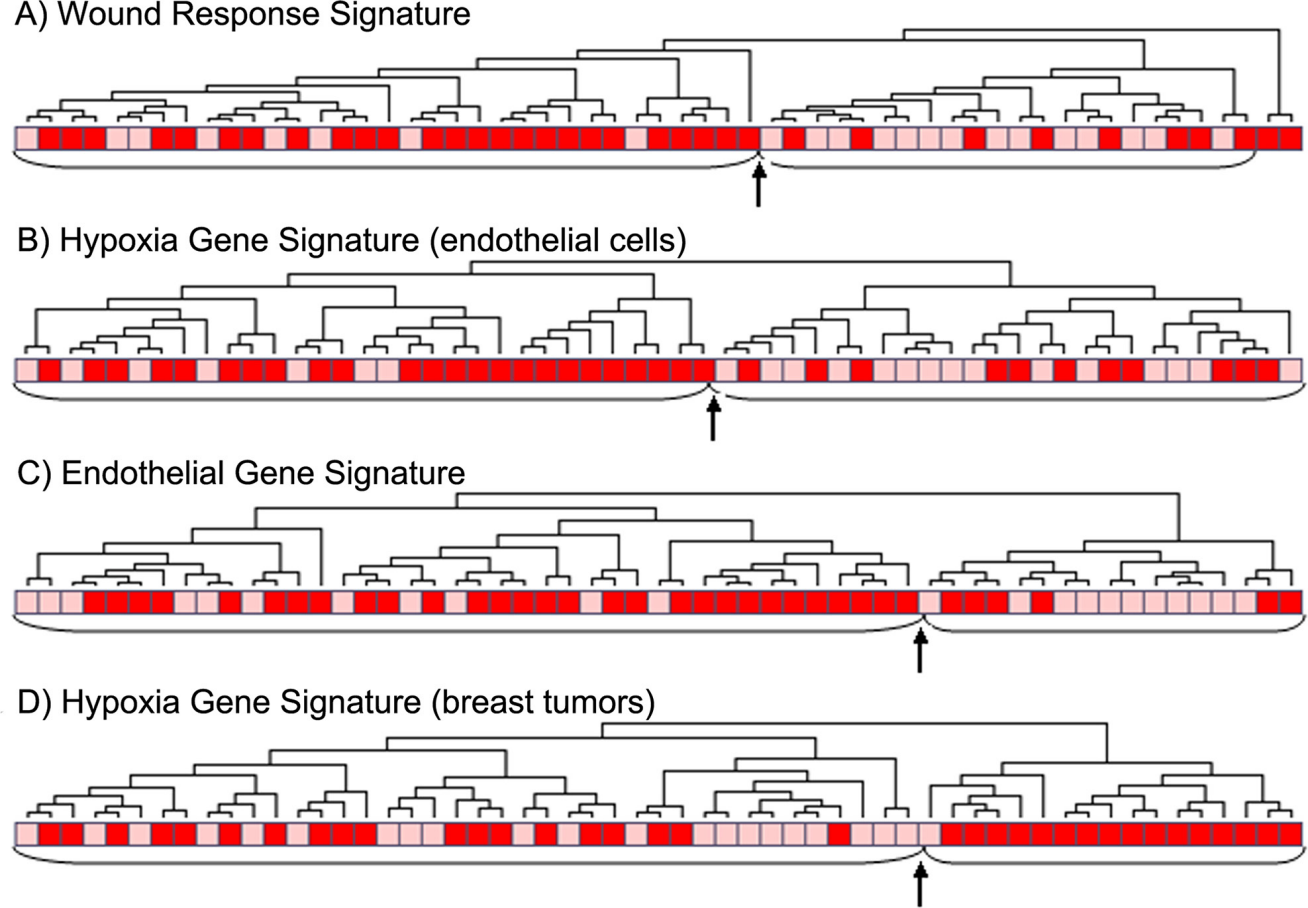
Clustering of tumors with (red) or without (pink) tumor necrosis using different gene signatures **A.** the wound response signature of 389 genes [29], *p* = 0.003, **B.** differentially expressed genes to hypoxia by HIF1A in vascular endothelial cells [20], 460 genes, *p* = 0.007, **C.** the endothelial gene signature of 28 genes [24], *p* = 0.008 and **D.** the hypoxia gene signature [21], 101 genes, *p* = 0.0009. (The arrow points to the border between two main clusters).

**Figure 4:**
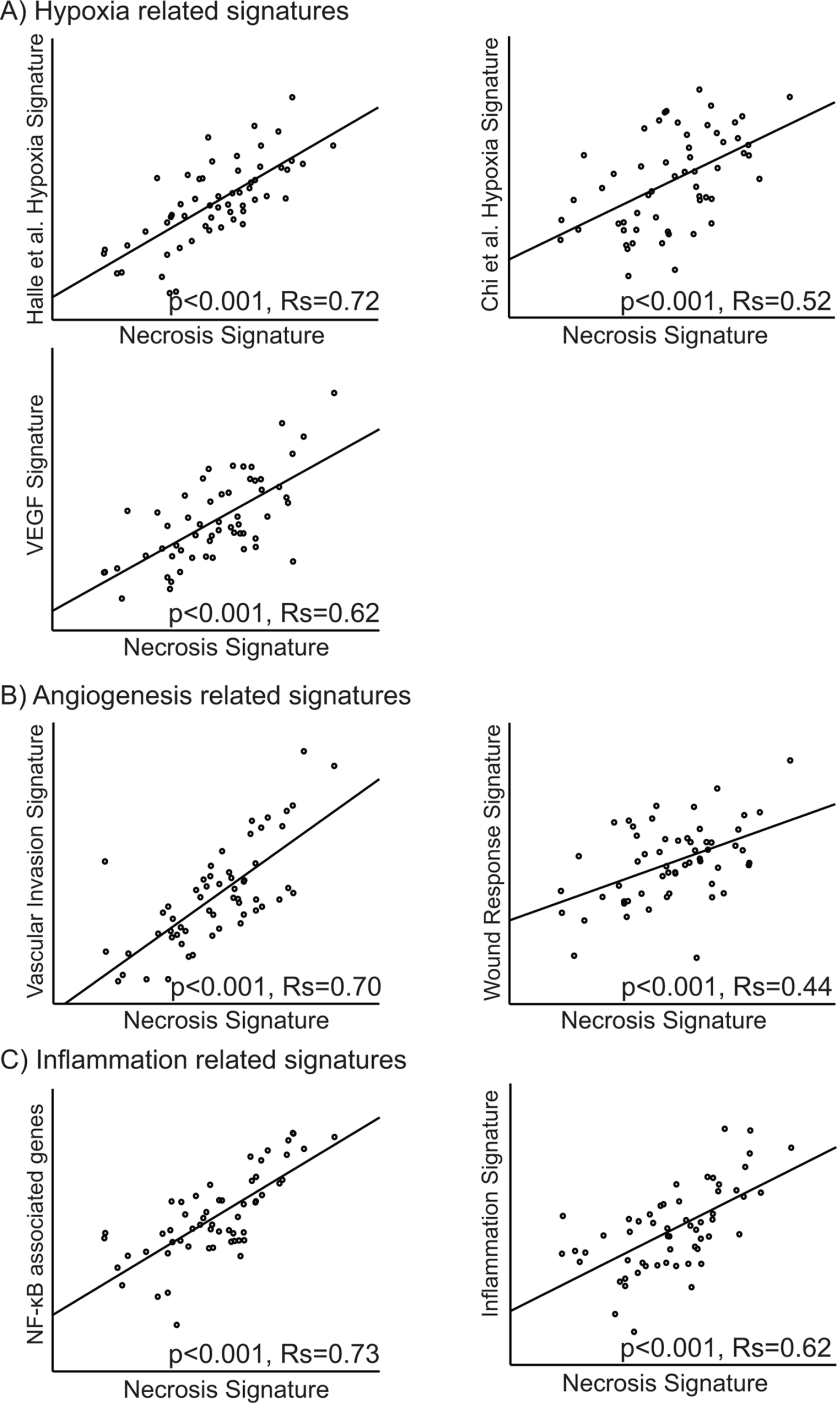
Correlation between the necrosis signature and signatures related to A. hypoxia [22, 23, 74], B. angiogenesis [29, 31] and C. inflammation [32, 33]. The Spearman rank correlation test was used for bivariate correlations

In our data, two of the main subgroups from hierarchical clustering of the hypoxia gene signatures from Manola and Nuyten [20, 21], were significantly associated with tumor necrosis (*p* = 0.013 and *p* = 0.0009, respectively) (Figure 3b and 3d). Further, three other gene signatures related to hypoxia were mapped to our data set [22–24], and correlations between our necrosis signature score and the applied hypoxia signature scores were explored. The tumor necrosis signature (TNS) was significantly correlated to all hypoxia signatures with R_s_ from 0.52–0.72 (Figure 4A).

The 38-gene necrosis signature was investigated in endometrial cancer cell lines exposed to hypoxic conditions. The necrosis signature showed a significantly higher score among the 8 biological replicates under hypoxia compared to normoxic conditions, reflecting tumor cell intrinsic responses (Wilcoxon Signed Rank test, related samples, *p* = 0.05) (Supplementary Table S3).

### Exploration of potential treatment targets in tumors with presence of necrosis

Genes differentially expressed between tumors with and without necrosis was applied in Connectivity Map analysis. Among 1309 small molecules represented in Connectivity Map, the drug signatures of Emetine and Cephaeline were among the top ranked drug signatures negatively correlated with the tumor necrosis signature, and may thus be potential inhibitors of tumor samples with necrosis (Table 4). These drugs have been reported to inhibit activation of HIF1A [25, 26], supporting HIF1A to play a role in tumors with necrosis. Also, signatures of PI3K/mTOR inhibitors were negatively correlated to the necrosis signature (Table 4). These drugs have been shown to inhibit HIF1A [27, 28], and PI3K and mTOR are here suggested as potential targets in cases with tumor necrosis.

**Table 4:** CMAP anlysis of 330 up-regulated genes (*p* < 0.024) and 390 down-regulated genes (*p* < 0.021) in necrotic tumors compared with non-necrotic tumor samples (according to *t*-test)

Perturbagen	*n*	Enrichment	*P*	Mechanism of action
Emetine	4	− 0.957	< 0.0001	Inhibitor of protein synthesis
LY-294002	61	− 0.595	< 0.0001	Inhibitor of PI3Ks
Sirolimus	44	− 0.507	< 0.0001	Inhibitor of mTOR
Wortmannin	18	− 0.605	< 0.0001	Inhibitor of PI3Ks
Quinostatin	2	− 0.993	0.0002	Inhibitor of PI3Ks
Cephaeline	5	− 0.788	0.0008	Inhibitor of protein synthesis

The perturbagens negatively connected with the genes with ***p***-value < 0.001

### Tumor necrosis is associated with markers of angiogenesis

Both in Series I (*n* = 57) and Series II (*n* = 286), necrosis was associated with vascular invasion (Table 1). Several angiogenesis-related genes like MMP1, MMP3, SFRP2, CXCL8, SERPINE1 and TNFAIP6 were up-regulated in tumors with necrosis, as identified among genes differentially expressed between tumor samples with and without necrosis (Table 2 and Supplementary Table S2).

Hierarchical clustering of the genes from the endothelial signature [24] in our data set gave a significant association between the resulting two clusters and necrosis status in the tumors (*p* = 0.015) (Figure 3c). Also, clustering of the wound response gene signature [29] showed a significant association between the resulting two clusters and tumor necrosis (*p* = 0.009) (Figure 3a). Two other gene signatures related to angiogenesis were mapped to our data set [29–31]. The correlations between these and the necrosis signature were explored, and the necrosis signature score was significantly correlated to both signature scores, with Spearman correlation coefficients, R_s_, of 0.70 and 0.44, respectively (Figure 4b). GSEA analysis showed several datasets related to angiogenesis to be up-regulated in samples with necrosis (Table 3).

In addition to gene expression analysis, the tumors in Series II have been investigated by IHC for the angiogenic factors VEGF-A, VEGF-C, VEGF-D and bFGF. Presence of tumor necrosis was significantly associated with increased expression of these angiogenic markers (Supplementary Table S4), supporting a relation between tumor necrosis and angiogenesis.

### Tumor necrosis is associated with markers of inflammation

By supervised analysis we found that multiple genes related to inflammatory processes and immune responses were significantly up-regulated in tumors with necrosis, such as chemokines (CXCL8, CCL20, CXCL1), metalloproteinases (MMP1, MMP3, MMP9) and proinflammatory genes (IL1B, PTGS2). In addition, numerous immunoglobulin genes were up-regulated in necrotic tumors (Table 2, Supplementary Table S2).

GO analysis showed significant enrichment (*p* < 0.0001) of gene sets reflecting various immune related responses as natural killer cell mediated immunity, immunity and defense, and granulocyte-mediated immunity (data not shown). By GSEA, several gene sets involved in inflammatory processes, among them different NF-κB gene sets, were enriched in tumors with necrosis (Table 3). The analysis indicated that genes under control of NF-κB, genes induced in activated dendritic cells, genes up-regulated in activated neutrophils, and genes induced by CCL20, IL1B and CXCL1, were up-regulated (Supplementary Table S2).

NF-κB related genes and a dendritic cell inflammation signature were mapped to our data set [32, 33]. The necrosis signature score was significantly correlated to both the NF-κB genes and the dendritic inflammation signature with R_s_ of 0.73 and 0.62, respectively (Figure 4C).

Series II had information on lymphocytic infiltration, and the association with necrosis was investigated. Tumors with presence of necrosis showed significant association with perivascular lymphocytic infiltration (Supplementary Table S5). In subgroup analyses, this association was present among endometrioid carcinomas only (*p* = 0.001), and not among non-endometroid tumors (*p* = NS) (data not shown).

### Tumor necrosis is associated with estrogen receptor negative tumors

Both Series I and II show tumor necrosis to be associated with eswwtrogen receptor negative tumors (Table 1). GSEA analysis supports this, showing estrogen responsive gene sets to be related to necrosis (Table 3).

## DISCUSSION

Tumor hypoxia, an important hallmark of aggressive cancers [1], is related to a more stimulatory microenvironment with increased angiogenesis and inflammation as co-existing responses [34, 35]. The presence of tumor necrosis, a morphologic marker of hypoxia, is associated with impaired prognosis in multiple malignant tumors [3–5]. Our previous studies have indicated a relationship between tumor necrosis and activated angiogenesis in endometrial carcinomas, as we found significantly increased vascular proliferation in tumors with necrosis [7]. However, less is known about gene expression programs and signaling pathways related to tumor necrosis in patient samples. In this study, we identified a 38-gene signature related to tumor necrosis in endometrial cancers. The necrosis gene signature showed associations with multiple features like hypoxia, angiogenesis and inflammation, and potentially novel treatment targets in the most aggressive tumors were suggested.

### Tumor necrosis and hypoxia

It is well established that tumor necrosis is initiated by tissue hypoxia during tumor progression due to rapid tumor growth and inadequate blood supply [36]. In line with this, we found that a panel of endometrial cancer cell lines grown under hypoxic conditions showed some up-regulation of the tumor necrosis signature derived from clinical samples, reflecting tumor cell intrinsic responses. Hypoxia is a marker of more aggressive tumors [18, 37, 38], and tumor necrosis was significantly associated with features such as high histologic grade, vascular invasion by tumor cells and reduced survival. In relation to treatment, hypoxia is thought to be a predictive marker for radiotherapy and chemotherapy response, since low oxygen levels are associated with less effective radiation treatment and reduced drug delivery [35, 39, 40].

In our study, drug signatures of Emetine and Cephaeline, known to inhibit HIF1A [25], were negatively associated with the tumor necrosis signature, further supporting hypoxia as an important feature of aggressive endometrial cancer. Also, PI3K/mTOR inhibitors could represent potential treatment for necrosis positive tumors according to our findings.

### Tumor necrosis and angiogenesis

We found that tumor necrosis and the necrosis related gene signature were associated with different angiogenic responses. Thus, tumors with necrosis were significantly linked to increased expression of multiple angiogenesis markers, such as IL1B, CXCL8, CXCL1, MMP1, MMP3, MMP9, SFRP2, PTGS2, BCL2A1, STC1, SOD2, SERPINE1, TNFRSF12A, FN1, ANGPTL4, ICAM1, and PTTG1. Common to the majority of these angiogenesis supporting genes is that they are induced by hypoxia and under transcriptional control of either HIF1A or NF-kB [41].

In contrast, HAND2 was found to be down-regulated in tumors with necrosis. A previous study demonstrated that HAND2 is required for proper vascular development, and HAND2 knockout mice demonstrated embryonic lethality [42]. Endothelial differentiation was unaltered, but endothelial cell patterning and smooth muscle cell differentiation was disrupted leading to abnormal vascular development. These findings suggest that HAND2 inactivation may lead to the development of dysfunctional and leaky vessels commonly seen in malignant tumors [42]. Also, a recent study on endometrial cancers showed HAND2 knock-out mice to get more pre-neoplastic alterations [43].

SFRP2 was especially increased among tumors with necrosis, being more than five-fold up-regulated. Recently, this protein was found to be overexpressed in the vasculature of 85% of human breast tumors and a novel stimulator of angiogenesis via a calcineurin dependent pathway [44]. Thus, tumor hypoxia might stimulate SFRP2 and trigger both NF-kB dependent pathways and multiple angiogenic factors [44, 45]. Interestingly, SEMA3E was 2.3-fold down-regulated in necrotic tumors, and this protein has also been involved in vascular patterning [46]. SEMA3E overexpression in a tumor xenograft model dramatically decreased the metastatic potential [47].

HIF1A is one of the major mediators of hypoxia induced VEGF expression [48]. Here, we found that tumors with necrosis showed significantly increased expression of angiogenic factors VEGF-A, VEGF-C and VEGF-D. bFGF expression is reported to be increased during inflammation and in necrotic cells, and elevated levels have been associated with better response to chemotherapy [49, 50]. In our study, we found bFGF protein levels to be increased in tumors with necrosis.

### Tumor necrosis and inflammation

Expression of NF-kB, a nuclear transcription factor involved in stress responses, is stimulated by tissue hypoxia [51]. Our data indicate that in cancers with necrosis, a substantial part of the up-regulated genes associated with inflammation is under transcriptional control of NF-kB. These include multiple factors such as CXCL8, CXCL1, MMP1, MMP9, VCAM1, ANGPTL4, ICAM1, and PLAU [52–54]. In support of this, a previous study indicated a potential role of NF-kB in endometrial cancer [55]. IL1B, an inducer of NF-kB [56, 57], was up-regulated two-fold, and significant enrichment of genes induced by IL1B was found by GSEA. The IL1 family itself is also under control of NF-kB [58], and this axis might represent a biological amplification mechanism. The inflammation amplifier reflect genes associated with cancer development [59], and several genes from the amplifiers were found up-regulated in necrotic tumors, like PTGS2, IL6, SERPINE1 and SOD2. Studies have reported high IL1 concentrations within the tumor microenvironment in association with aggressive tumors [60]. The correlation between NF-kB related genes and our necrosis signature support this.

### Tumor necrosis and estrogen receptor expression

Estrogen signaling is important in the biology of endometrial cancer, and there are striking phenotypic differences between estrogen receptor positive and negative tumors [61]. Here, ER negative cancers were significantly associated with tumor necrosis. We observed by GSEA a significant enrichment of genes in tumors with necrosis that are also up-regulated in estrogen receptor negative breast cancers [16].

Loss of estrogen receptor expression is observed to activate NF-kB [62], and ESR1 and NF-kB are known to mutually antagonize each other [63]. Correspondingly, estrogen and progesterone receptors have the ability to block NF-kB activation [64]. It is not clear, however, whether NF-κB activation is thus an intrinsic property of ER negative endometrial cancers, or is activated secondary to tumor necrosis, or a combination.

Thus, our findings derived from patient samples indicate that tumor necrosis is associated with multiple co-existing high-grade features and responses such as tumor angiogenesis and tumor inflammation. This might indicate a potential co-regulation of interacting tumor programs linked to hypoxia in these tumors. The findings are consistent in different analysis approaches. However, further studies are needed to expand on the mechanistic links that might be involved and treatable.

## CONCLUSION

Presence of tumor necrosis was reflected by increased expression of multiple gene sets and pathways related to tumor-microenvironment interactions, such as tissue hypoxia, angiogenesis and inflammation, indicating complex cross-talk as well as potentially novel biomarkers and therapy targets. Tumor necrosis in endometrial cancers is associated with ER negative tumors and NF-kB activation. As indicated by our data, HIF1A, NF-kB and PI3K/mTOR might be potential targets in aggressive endometrial cancers with presence of tumor necrosis.

## MATERIALS AND METHODS

### Patient series

Microarray and qPCR experiments were performed on a prospectively collected explorative series of fresh frozen specimens (Series I). Clinico-pathologic correlates and outcome were also studied in this series and in a retrospective series for validation (Series II). Series I: During 2001–2003, 57 cases of endometrial cancer (median age 63.0 years) were prospectively collected at the Department of Gynecology and Obstetrics, Haukeland University Hospital, University of Bergen, Norway, as previously described. [19] Series II: A population based series including all endometrial tumors diagnosed in Hordaland County (approximately 500,000 inhabitants, 10% of the Norwegian population) during 1981–1990, containing 316 formalin fixed and paraffin embedded tumors, was used as validation series [9].

### Pathology

The following clinico-pathological variables were included for both series: histologic type, histologic grade, myometrial infiltration, estrogen and progesterone receptor status, mitotic count, necrosis, vascular invasion, and FIGO stage as previously reported [7, 9, 65]. Briefly, tumor necrosis was defined as areas of necrotic tumor cells immediately adjacent to viable tumor tissue [66]. The prospective series contained information on tumor category according to a 29 gene signature (cluster I and II) [19]. In addition, the retrospective series (Series II) had information on morphologic cancer type I/II [67], perivascular lymphocytic infiltration (PLI), tumor infiltrating lymphocytes (TIL) [68], and data on VEGF-A, VEGF-C, VEGF-D and bFGF expression by immunostaining [9].

### Follow-up

For Series I, patients were followed from the time of primary surgery until September, 2008 or until death. Median follow-up time for survivors was 5.1 years (range 0.9 – 7.0 years). All events (14 recurrences, 7 deaths from cancer) were recorded, and deaths due to other causes were censored in survival analyses. No patient was lost to follow-up. For Series II, of all 316 patients diagnosed with endometrial carcinoma in this population based series, there were 286 cases available for this study. Patients were followed from the time of primary surgery until death or last follow up, August 2004. Median follow-up time for the survivors was 17 years (range 10–23 years); 74 patients died from endometrial cancer. Cases without evaluable staining were excluded from the analysis of immunohistochemical markers.

### Microarray analysis

Total RNA was extracted from biopsies with at least 50% (usually > 80%) tumor content using the RNeasy minikit (Qiagen, Valencia, CA). Quality and yield were assessed by agarose electrophoresis, Agilent Bioanalyser 2100 (Agilent Technologies, Santa Clara, CA) and spectrophotometry. T7 RNA polymerase promoter containing cDNAs and Cy3 and Cy5 labeled cRNAs were generated and hybridized to Agilent 21k and 22k microarrays as described earlier [19, 69, 70]. All microarrays were scanned and features extracted using the Agilent Microarray Scanner Bundle (Agilent). Data was processed as described [19, 30]. This dataset has been deposited in the GEO DataSets, GSE14860.

### Differential gene expression

Significance analysis of microarrays (SAM) [71] was applied to look for genes differentially expressed between samples labeled necrotic positive (N+) and negative (N–). For the necrosis signature, a requirement of at least 2-fold change was applied together with a false discovery rate (FDR) of ≤ 0.06. For hypothesis genes, collected from the literature, SAM with FDR < 0.2 and fold change of 1.5 were used.

### Real-time PCR using taqman low density array format

For validation of microrray data, mRNA levels of a subset of genes were analyzed using Taqman low density arrays (TLDA) for real-time qPCR (Applied Biosystems, Foster City, CA). cDNA synthesis and PCR protocols were performed as previously described [72]. To confirm gene expression data from microarray analysis, 10 of the 38 genes from the SAM signature were analyzed by qPCR.

### Predictors of necrosis using fixed gene sets

Leave one out cross validation (LOOCV) was used to test the predictability of labels based on presence (N+) or absence (N−) of necrosis, using all except one sample as training data in each round. Cross validation was performed with the list of genes from SAM analysis fixed. Additionally, a condensed predictor was constructed from the SAM list genes in order to investigate if the same or better predictive power could be obtained with fewer genes in the predictive model. To construct this condensed predictor, techniques from machine learning called forward selection and backward elimination was applied [73]. Cross validation was then performed with this condensed predictor gene set. A diagonal linear discriminant was used as prediction model.

### Clustering of samples using thematic gene sets

Different gene signatures related to hypoxia, angiogenesis and inflammation were used to investigate relations to tumor necrosis. The hypoxic gene sets were Manalo's signature containing differentially expressed genes in vascular endothelial cells exposed to hypoxia compared to normoxic endothelial cells [20] and Nuyten's hypoxia gene signature containing 123 genes generated from breast tumors [21]. For angiogenesis signatures, genes in the wound response (core serum response) signature [29] and in an endothelium signature [24] were mapped to our data set. As relatively few of the genes in the endothelial signature were found in our microarray data (28 out of 64), an expansion of the signature was carried out by including genes with significant correlation (*r* > 0.65) to one of the 28 signature genes. This gave an expanded signature of 468 genes.

Subsequently, the samples were clustered (hierarchical clustering, average linkage WPGMA, Pearson's correlation) based on the expression levels of the signature genes. The clustering of samples was tested for association to necrosis status (N+ and N−).

### Gene signatures related to the necrosis signature

Different signatures were used to investigate how the necrosis gene signature associates with hypoxia, angiogenesis and inflammation. Three hypoxia gene signatures have been used. Chi et al. constructed a gene signature showing the hypoxic response in mammary and renal tubular epithelial cells [22]. The hypoxia signature from Halle et al. was created for cervical cancer based on cell culture experiments [23]. The VEGF signature identifies a compact *in vivo* hypoxia signature highly expressed in metastatic breast tumors [74]. Two signatures in relation to angiogenesis were used: the vascular invasion signature constructed from endometrial tumors [30, 31] and the wound response signature [29]. For inflammation, a signature of NF-κB-associated genes and an inflammation signature generated from dendritic cells were used [32, 33].

For correlation between signatures, summarized signature scores were calculated [75]. For the necrosis signature, the vascular invasion signature, the hypoxia signature from Chi et al. and the inflammation signature, summarized expression values for the down-regulated genes were subtracted from the sum of expression values for the up-regulated genes. For the wound response signature, a summary expression signature was generated for the activated genes. For the hypoxia signature from Halle et al., the VEGF signature and the NF-κB-regulated genes, a mean expression value from the expression values for the signature genes was calculated, as applied in the studies where the signatures were identified.

### Gene ontology (GO) analysis

The gene expression tools on the PANTHER (Protein ANalysis THrough Evolutionary Relationships) website was applied to the complete gene set (http://www.pantherdb.org/tools/genexAnalysis.jsp). Bonferroni correction for multiple testing was applied.

### Gene set enrichment analysis (GSEA)

Our data set was investigated based on gene sets available through MSigDB (http://www.broadinstitute.org/gsea/msigdb). Also, gene sets from the literature were constructed in GSEA and applied to our data set.

### Hypoxia stimulation of endometrial cancer cell lines

Endometrial cancer cell lines KLE, RL95-2, AN3 CA, HEC-1-A (Manassas, Virginia, USA) and MFE-280, MFE-296, EFE-184 (German Collection of Microorganisms and Cell Cultures, DSMZ, Braunschweig, Germany) were cultured, without technical replicates, in media recommended by the suppliers. The cells were incubated for 18 hours in normoxia (37^°^C, 5% CO_2_) or hypoxia (37^°^C, 5% CO_2_, 1.5% O_2_). The cells were harvested using 350 μl TRK Lysis Buffer per well from the E.Z.N.A.® Total RNA Kit I, and total RNA was purified in accordance with the manual (Omega Bio-Tek, Norcross, Georgia, USA). Quality and yield of the RNA samples were determined by agarose gel electrophoresis, Agilent Bioanalyzer 2100 and spectrophotometry. Gene expression was further investigated by microarray analysis as described above.

### Connectivity map

The correlation between the global expression pattern for patients with tumor necrosis and drug signatures was assessed in Series I by the Connectivity Map database [76]. Genes differentially expressed between tumor subsets with necrosis present and absent were included in the signature as the basis for the analyses in Connectivity Map. This gene list included 330 up- and 390 down- regulated genes.

### Statistical analysis

Statistical analyses were performed with the IBM SPSS Statistic version 21 (SPSS Inc., Chicago, IL). Linear associations between two continuous variables were evaluated by linear regression analysis and Spearman´s correlation. Univariate survival analyses were performed using the Kaplan-Meier method (log-rank significance test), and necrosis score was dichotomized based on the median. Associations between different categorical variables were assessed by Pearson's chi-square test. Wilcoxon Signed Rank test was used for comparing two related samples. Probability of < 0.05 was considered statistically significant.

## SUPPLEMENTARY FIGURE AND TABLES


