# Supplementary material for: Tumor necrosis is an important hallmark of aggressive endometrial cancer and associates with hypoxia, angiogenesis and inflammation responses
Source: Oncotarget. 2015 Oct 14;6(37):39676–91. doi: 10.18632/oncotarget.5344 (PMC4741854; doi:10.18632/oncotarget.5344)
Supplement: Supplementary file 1 [file oncotarget-06-39676-s001.pdf]

SUPPLEMENTARY FIGURE AND TABLES

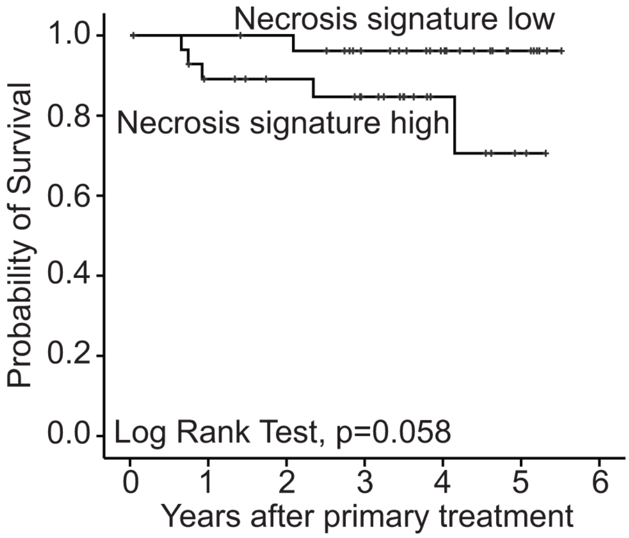

Supplementary Figure S1: Estimated survival among patients in Series I (*N* = 57) according to necrosis signature.

Supplementary Table S1: Clustering of the SAM genes in relation to clinico-pathologic variables in Series I ($N = 57$)

		Cluster 1	Cluster 2	p*
		n (%)	n (%)	
Necrosis	Absent	8 (36)	14 (64)	0.0001
	Present	30 (86)	5 (14)	
Histologic type	Endometrioid	32 (63)	19 (37)	0.07
	Non-endometrioid	6 (100)	0 (0)	
Histologic grade	Grade 1 and 2	27 (61)	17 (39)	NS
	Grade 3	11 (85)	2 (15)	
Type II cancer	No	23 (58)	17 (42)	0.024
	Yes	15 (88)	2 (12)	
Estrogen receptor†	Positive	24 (59)	17 (41)	0.037
	Negative	14 (89)	2 (11)	
Progesterone receptor†	Positive	26 (59)	18 (41)	0.026
	Negative	12 (92)	1 (8)	
Mitoses‡	Low	27 (64)	15 (36)	NS
	High	11 (73)	4 (27)	
Vascular invasion	Absent	19 (54)	16 (46)	0.012
	Present	19 (86)	3 (14)	
Myometrial infiltration	< 50%	19 (66)	10 (34)	NS
	≥ 50%	19 (68)	9 (32)	
FIGO stage	I/II	30 (63)	18 (37)	NS
	III/IV	8 (89)	1 (11)	
Aggressive cluster [1]	No	13 (45)	16 (55)	0.0004
	Yes	25 (89)	3 (11)	

*Chi-square test, two-sided
†Cut-point median
‡Cut-point upper quartile

Supplementary Table S2: Genes generated by SAM (FDR < 0.20, FC > 1.5) being differentially expressed between necrotic and non-necrotic tumors in relation to hypoxia, angiogenesis, inflammation and NF-κB binding sites

Hypoxia related genes

Gene symbol	Gene name
CXCL8	Chemokine (C-X-C motif) ligand 8
SERPINE1	Serpin Peptidase Inhibitor, Clade E, Member 1
IL6	Interleukin 6 (Interferon, Beta 2)
HCAR3	Hydroxycarboxylic Acid Receptor 3
PTGS2	Prostaglandin-Endoperoxide Synthase 2 (COX2)
SLC2A3	Solute Carrier Family 2 (Facilitated Glucose Transporter), Member 3
STC1	Stanniocalcin 1
LOX	Lysyl Oxidase
ANGPTL4	Angiopoietin-Like 4
SOD2	Superoxide Dismutase 2, Mitochondrial
ELL2	Elongation Factor, RNA Polymerase II, 2
LDHA	Lactate Dehydrogenase A
ADM	Adrenomedullin
ERO1L	ERO1-Like (S. Cerevisiae)
BRCA2	Breast Cancer 2, Early Onset
DDIT4	DNA-Damage-Inducible Transcript 4
TFRC	Transferrin Receptor (P90, CD71)
ELL2	Elongation Factor, RNA Polymerase II, 2
CTSF	Cathepsin F
FOS	V-Fos FBJ Murine Osteosarcoma Viral Oncogene Homolog

Angiogenesis related genes

Gene symbol	Gene name
MMP1	Matrix metallopeptidase 1 (interstitial collagenase)
SFRP2	Secreted frizzled-related protein 2
CXCL8	Chemokine (C-X-C motif) ligand 8
SERPINE1 (PAI1)	Serpin peptidase inhibitor, clade E, member 1
IL6	Interleukin 6 (interferon, beta 2)
CXCL1	Chemokine (C-X-C motif) ligand 1 (melanoma growth stimulating activity, alpha)
MMP3	Matrix metallopeptidase 3 (stromelysin 1, progelatinase)
FN1	Fibronectin 1
BCL2A1 (Bfl1)	BCL2-related protein A1
PTGS2 (COX2)	Prostaglandin-endoperoxide synthase 2
IL1B	Interleukin 1, beta
STC1	Stanniocalcin 1
MMP9	Matrix metallopeptidase 9

(*Continued*)

Angiogenesis related genes

Gene symbol	Gene name
ANGPTL4	Angiopoietin-like 4
TNFRSF12A	Tumor necrosis factor receptor superfamily, member 12A
ICAM1	Intercellular adhesion molecule 1 (CD54)
SOD2	Superoxide dismutase 2, mitochondrial
CCL20	Chemokine (C-C motif) ligand 20
ADM	Adrenomedullin
PTTG1	Pituitary tumor-transforming 1
JUN	V-jun sarcoma virus 17 oncogene homolog (avian)
HAND2	Heart and neural crest derivatives expressed 2
ANGPTL1	Angiopoietin-like 1
SERPINA5 (PCI)	Serpin peptidase inhibitor, clade A, member 5

Inflammation related genes

Gene symbol	Gene name
MMP1	Matrix metallopeptidase 1 (interstitial collagenase)
IGLV1–51	Immunoglobulin lambda variable 1–51
CXCL8	Chemokine (C-X-C motif) ligand 8
TNFAIP6	Tumor necrosis factor, alpha-induced protein 6
IGKV4–1	Immunoglobulin kappa variable 4–1
IGKV6–21	Immunoglobulin kappa variable 6–21
CXCL1	Chemokine (C-X-C motif) ligand 1 (melanoma growth stimulating activity, alpha)
MMP3	Matrix metallopeptidase 3 (stromelysin 1, progelatinase)
IGHA1	Immunoglobulin heavy constant alpha 1
CCL20	Chemokine (C-C motif) ligand 20
IGLV5–37	Immunoglobulin lambda variable 5–37
IGLV3–19	Immunoglobulin lambda variable 3–19
IGKV1–33	Immunoglobulin kappa variable 1–33
IL1RN	Interleukin 1 receptor antagonist
IGHV3–48	Immunoglobulin heavy variable 3–48
IGHV3–11	Immunoglobulin heavy variable 3–11
BCL2A1	BCL2-related protein A1
IL6	Interleukin 6
IL1B	Interleukin 1, beta
PTGS2/COX2	Prostaglandin-endoperoxide synthase 2
MMP9	Matrix metallopeptidase 9

(*Continued*)

Genes with NF-κB binding sites	
**Gene symbol**	**Gene name**
CXCL8	Chemokine (C-X-C motif) ligand 8
CCL20	Chemokine (C-C motif) ligand 20
CXCL1	Chemokine (C-X-C motif) ligand 1 (melanoma growth stimulating activity, alpha)
IL1RN	Interleukin 1 receptor antagonist
PTGS2/COX2	Prostaglandin-endoperoxide synthase 2
BCL2A1	BCL2-related protein A1
IL1B	Interleukin 1, beta
IL6	Interleukin 6
SOD2	Superoxide dismutase 2, mitochondrial
MMP9	Matrix metallopeptidase 9
SLC2A5	Solute carrier family 2
TNC	Tenascin C
ICAM1	Intercellular adhesion molecule

Supplementary Table S3: The tumor necrosis signature score in endometrial cancer cells cultured in hypoxia (for 18 hours) versus normoxia

Cell line	Necrosis Signature Score		*P*- value*
	Normoxia	Hypoxia	
AN3CA	98.93	100.83	0.05
RL95–2	100.00	98.93	
EFE-184	98.57	101.14	
MFE-296	99.50	99.70	
HEC-1-A	100.48	101.78	
HEC-1-B	101.94	102.36	
KLE	97.41	98.86	
MFE-280	96.60	97.36	

*Related samples, Wilcoxon Signed Rank test

Supplementary Table S4: Associations between tumor necrosis and VEGF-A, VEGF-C, VEGF-D and bFGF immunostaining in Series II, *N* = 286

	Necrosis absent	Necrosis present	*P* value*
	N (%)	*N* (%)	
**VEGF-A**†			**0.003**
low	103 (46)	122 (54)	
high	11 (22)	38 (78)	
**VEGF-C**‡			**0.019**
low	59 (50)	60 (50)	
high	55 (36)	100 (64)	
**VEGF-D**‡			**0.018**
low	65 (49)	68 (51)	
high	49 (35)	92 (65)	
**bFGF**			**0.007**
low	55 (52)	50 (48)	
high	60 (36)	108 (54)	

*Chi-square test, two-sided
†Cut-point upper quartile.
‡Cut-point median.

Supplementary Table S5: Associations between tumor necrosis and lymphocytic infiltration in Series II, *N* = 286

	Necrosis absent	Necrosis present	*P* value*
	N (%)	*N* (%)	
**Perivascular lymphocytic infiltration**			**0.001**
No	99 (47)	110 (53)	
Yes	20 (26)	57 (74)	
**Tumor infiltrating lymphocytes**			**NS**
Absent	92 (44)	117 (56)	
Weak	14 (41)	20 (59)	
Brisk	13 (30)	30 (70)	

*Chi-square test, two-sided

REFERENCES

1. Bokhman JV. Two pathogenetic types of endometrial carcinoma. Gynecologic oncology. 1983; 15:10–17.
2. Salvesen HB, Carter SL, Mannelqvist M, Dutt A, Getz G, Stefansson IM, Raeder MB, Sos ML, Engelsen IB, Trovik J, Wik E, Greulich H, Bo TH, et al. Integrated genomic profiling of endometrial carcinoma associates aggressive tumors with indicators of PI3 kinase activation. Proc Natl Acad Sci U S A. 2009; 106:4834–4839.
3. White JR, Harris RA, Lee SR, Craigon MH, Binley K, Price T, Beard GL, Mundy CR, Naylor S. Genetic amplification of the transcriptional response to hypoxia as a novel means of identifying regulators of angiogenesis. Genomics. 2004; 83:1–8.
4. Chi JT, Wang Z, Nuyten DS, Rodriguez EH, Schaner ME, Salim A, Wang Y, Kristensen GB,

Helland A, Borresen-Dale AL, Giaccia A, Longaker MT, Hastie T, et al. Gene expression programs in response to hypoxia: cell type specificity and prognostic significance in human cancers. PLoS medicine. 2006; 3:e47.

5. Ghosh S, Spagnoli GC, Martin I, Ploegert S, Demougin P, Heberer M, Reschner A. Three-dimensional culture of melanoma cells profoundly affects gene expression profile: a high density oligonucleotide array study. Journal of cellular physiology. 2005; 204:522–531.
6. Gerritsen ME, Tomlinson JE, Zlot C, Ziman M, Hwang S. Using gene expression profiling to identify the molecular basis of the synergistic actions of hepatocyte growth factor and vascular endothelial growth factor in human endothelial cells. British journal of pharmacology. 2003; 140:595–610.
7. Lerebours F, Vacher S, Andrieu C, Espie M, Marty M, Lidereau R, Bieche I. NF-kappa B genes have a major role in inflammatory breast cancer. BMC cancer. 2008; 8:41.
8. Rossi M, Sharkey AM, Vigano P, Fiore G, Furlong R, Florio P, Ambrosini G, Smith SK, Petraglia F. Identification of genes regulated by interleukin-1beta in human endometrial stromal cells. Reproduction. 2005; 130:721–729.
9. Van't Veer LJ, Dai H, van de Vijver MJ, He YD, Hart AA, Mao M, Peterse HL, van der Kooy K, Marton MJ, Witteveen AT, Schreiber GJ, Kerkhoven RM, Roberts C, et al. Gene expression profiling predicts clinical outcome of breast cancer. Nature. 2002; 415:530–536.
10. Tang S, Han H, Bajic VB. ERGDB: Estrogen Responsive Genes Database. Nucleic acids research. 2004; 32:D533–536.
